# The role of health workers in Kenya’s Net-Zero transition: a Mixed-Methods study on healthcare system climate change mitigation and adaptation

**DOI:** 10.1093/oxfclm/kgaf026

**Published:** 2025-12-01

**Authors:** Iris Martine Blom, Melvine Anyango Otieno, Marie-Claire Wangari, Agan Leonard, Winslet Mwende, Naomi Wanjiku Gitau, Iain Cross, Anita Berlin, Andy Haines, Sarah Whitmee

**Affiliations:** Centre on Climate Change and Planetary Health, London School of Hygiene and Tropical Medicine, London, WC1E 7HT, United Kingdom; School of Environmental Sciences and Natural Resource Management, University of Eldoret, Eldoret, 30100, Kenya; Kenya Medical Association, Nairobi, 00100, Kenya; Department of Environmental Sciences, University of Eldoret, Eldoret, 30100, Kenya; Moi University, Kipkenyo, Cheptiret, 30100, Kenya; Moi University, Kipkenyo, Cheptiret, 30100, Kenya; Centre for Excellence in Learning and Teaching, London School of Hygiene and Tropical Medicine, London, WC1E 7HT, United Kingdom; Queen Mary University of London, London, E1 4NS, United Kingdom; Centre on Climate Change and Planetary Health, London School of Hygiene and Tropical Medicine, London, WC1E 7HT, United Kingdom; Centre on Climate Change and Planetary Health, London School of Hygiene and Tropical Medicine, London, WC1E 7HT, United Kingdom

**Keywords:** climate change, planetary health, health systems, sustainability, resilience, mitigation, adaptation, health workforce, health workers, education

## Abstract

Climate change presents a critical challenge to healthcare systems, particularly in low- and middle-income countries like Kenya. Health workers are key to leading the transition toward a sustainable, climate-resilient healthcare system. This mixed-methods study explores the perceptions, knowledge, and roles of Kenyan health workers in mitigation and adaptation in healthcare. An online questionnaire, completed by 118 health workers, explored their understanding of climate change’s impacts on health, the healthcare system’s role in emissions reduction and adaptation, and current practices. A subsequent focus group discussion delved deeper into the identified themes, with a particular focus on education of health workers to support climate action. The findings reveal that while health workers are aware of the health risks posed by climate change, financial limitations and insufficient training present significant barriers to the implementation of sustainable practices. The focus group emphasized the need for practical, context-specific education to equip health workers with actionable knowledge and skills, alongside fostering emotional resilience and ethical leadership. Key recommendations include co-creating educational programs with communities and health workers, integrating climate-health modules into curricula, and leveraging innovative approaches such as peer-led workshops and social media campaigns. These insights underscore the transformative potential of education in empowering health workers to lead Kenya’s transition to a sustainable, climate-resilient healthcare system.

## Introduction

Climate change presents an unprecedented challenge to global healthcare systems. It is increasingly recognized as the largest health threat of the 21st century, exacerbating existing health issues and introducing new risks [1]. Healthcare systems, responsible for about 5% of global greenhouse gas (GHG) emissions, are both a contributor to the crisis and heavily affected by its consequences [2]. Most of these emissions come from healthcare systems in high income countries and, going forward, low-emitting countries will have important policy choices about GHG emitting sectors including healthcare [1]. As healthcare systems aim to manage the adverse impacts of climate change, they must simultaneously adapt to the change that cannot be prevented and mitigate their environmental footprint.

Low- and middle-income countries (LMICs), including Kenya, are disproportionately vulnerable to the health impacts of climate change. Kenya is facing both direct health effects—such as increased frequency of heatwaves and changing patterns of infectious diseases—and indirect effects, including reduced access to essential services and infrastructure [3]. In response, Kenya has committed to transitioning its healthcare system toward a resilient system with net-zero emissions by 2030, as part of the World Health Organization’s (WHO) United Nations Framework Convention on Climate Change 26^th^ Conference of Parties (UNFCCC COP26) Health Programme in 2021 [4]. Following Kenya’s National Climate Change Action Plan (NCCAP) which recognized the importance of integration of climate change into all sectors including health, Kenya identified key strategic actions including developing education programs to empower communities, enhancing disaster preparedness, and strengthening resilience against climate-induced health challenges, and integrating climate change into cross-sector curricula at all levels including for the health workforce [5].

Kenya’s health professionals are recognized by decision-makers as central stakeholders in the country’s transition to a climate resilient, net-zero healthcare system [6, 7]. Their role extends beyond patient care to actively influencing the planning, implementation, and evaluation of climate adaptation and mitigation strategies. Interviews conducted in Kenya in 2023 with key stakeholders in the healthcare system transformation affirm that health workers are pivotal in guiding sustainable practices at every level of healthcare delivery, ensuring that interventions are feasible, impactful, and aligned with national climate objectives [7]. Beyond implementation, the active engagement of these health workers is crucial for the design of solutions, the development of national sustainable healthcare policies, and the generation of localized data to inform climate actions. This mirrors findings from other contexts, such as in England’s “Greener NHS” programme, where health workers have been instrumental in leading low-carbon initiatives, and in Australia, where health professionals underlined their role in implementation towards sustainable, climate-resilient healthcare [8, 9]. Health professionals’ capacity to drive change and willingness to engage are indispensable for achieving Kenya’s ambitious climate targets within its healthcare system.

In this manuscript, we describe health workers’ perceptions of their roles and contributions to Kenya’s net-zero, resilient and sustainable healthcare transition. Through a mixed-method approach—including a questionnaire and a focus group discussion with health workers—we explore integrating climate change mitigation and adaptation into routine healthcare delivery. By focusing on the perceptions of health workers, we provide a first step towards understanding how they can best be supported to drive the necessary transformation toward a resilient, sustainable healthcare system.

## Methods

This study employed a mixed-methods approach to investigate the roles and perceptions of health workers in Kenya’s transition to a net-zero healthcare system. The study was conducted in two phases: (i) a structured questionnaire aimed at capturing baseline knowledge, attitudes, and practices of health workers regarding climate change mitigation and adaptation, and (ii) a focus group discussion, informed by the outcomes of the questionnaire, further explored barriers, opportunities, and actionable strategies for health workers to contribute to sustainable healthcare practices.

### Study setting and participants

The online study targeted health workers and university students in Kenya, including medical doctors, nurses, pharmacists, community health workers, dentists, and those training in these professions. For the purpose of this study, the term ‘health workers’ is used inclusively to refer to both practicing professionals and students, acknowledging their active roles in healthcare delivery through clinical placements and community engagement. Participants were recruited through outreach to professional and student health associations, representing the diverse healthcare workforce across the country. These associations were identified using the authors’ prior knowledge, professional networks, and publicly available information, ensuring representation from a range of healthcare institutions, including public hospitals, private facilities, and community health centres.

For the questionnaire, convenience sampling was employed based on participants’ availability and willingness to participate. The questionnaire was disseminated through existing association communication channels and public social media platforms. As a result of this sampling method, response rates could not be calculated. Convenience sampling was used in this study to efficiently explore this area for the first time, addressing challenges such as transnational communication and recruitment constraints.

For the focus group, purposive sampling was used to select representatives from 12 professional healthcare associations and their student or young professional networks. Each association was invited to nominate one representative to convey their collective perspectives, and a total of seven representatives were ultimately nominated and participated in the discussion, representing community health workers, dentists, pharmacists, nurses, medical doctors, family physicians, and pharmacy and medical students. Focus groups were chosen as the primary method for this phase due to their ability to facilitate group interaction, generate rich and diverse insights, support exploratory research by enabling participants to build upon each other’s ideas, and provide a deeper understanding of collective perspectives and dynamics, ensuring representation from key stakeholders and offering a comprehensive initial exploration of educational and policy needs [10].

### Phase 1: Questionnaire

A structured questionnaire was distributed online to health workers across Kenya to assess knowledge, perceptions, and current engagement in climate change-related mitigation and adaptation practices. The questionnaire (Appendix 1) included both closed and open-ended questions designed to assess various aspects of healthcare professionals’ perceptions and practices related to climate change. The questionnaire was developed based on a review of relevant literature and drafted collaboratively by the research team. It was refined through feedback from a pilot group of 10 Kenyan healthcare professionals, ensuring clarity, cultural relevance, and alignment with the study’s objectives. Questions addressed the following topics: participants’ awareness of climate change and its health impacts; their understanding of healthcare’s contribution to greenhouse gas emissions; existing transformation efforts within healthcare settings; barriers and opportunities to implementing climate change mitigation and adaptation strategies; and participants’ willingness to engage in healthcare system transformation.

### Phase 2: Focus group discussion

The focus group discussion, conducted after the questionnaire, was designed to delve deeper into the themes that emerged from this initial exploration. The questionnaire provided a broad overview of healthcare workers’ knowledge, perceptions, and practices related to climate change, highlighting education as a critical gap. Building on these findings, the focus group further explored education by concentrating the current understanding and perception of climate change within respective healthcare professional groups, the role of health workers in climate mitigation and adaptation efforts (including an exploration of power dynamics in driving change and implementing educational initiatives), an exploration of knowledge and training needs regarding sustainable and resilient healthcare (with attention to local knowledge systems and contextualised educational approaches), and barriers and opportunities for implementing climate change education within healthcare (Appendix 2).

To ensure a culturally sensitive and inclusive discussion, two facilitators were present. One (IMB) led the discussion, while the second (MO) observed cultural nuances, monitored participant engagement, and provided input or clarifications to maintain sensitivity. The second facilitator also provided feedback to refine the analysis, supporting a safe and inclusive environment for all participants. The focus group was conducted via Zoom due to geographical constraints, lasting approximately two and a half hours. It was held in English, which was the preferred and professionally appropriate language for all participants, as confirmed during recruitment; no one was excluded on the basis of language. All discussions were audio-recorded with participants’ consent and transcribed verbatim for analysis.

### Data analysis

Data from the questionnaire were analysed using descriptive statistics to summarize respondents’ knowledge, attitudes, and practices concerning climate change mitigation and adaptation. Categorical variables were summarized as frequencies and percentages, while continuous variables were presented as means and standard deviations. Responses to open-ended questions were thematically coded to identify recurring themes related to barriers and opportunities for action.

Transcripts from the focus group discussion were analysed using thematic analysis. Initial coding was performed using NVivo software to identify major themes, followed by a second round of analysis to refine and categorize these themes. Key findings were triangulated with the results from the questionnaire to provide a comprehensive understanding of the health workers’ perceptions and roles in the net-zero healthcare transition.

### Ethical considerations

The proposal for this research was approved by the Research Ethics Committee of the London School of Hygiene & Tropical Medicine (Ref. 28210) and the Kenya Medical Research Institute (KEMRI, Ref. 4662), and licensed by the National Commission for Science, Technology and Innovation (NACOSTI, Ref. 519115 and extension Ref. 285069). Written informed consent was obtained through the questionnaire form and ahead of the focus group from all participants prior to their participation in the study. Confidentiality was maintained throughout the research process. All participants were informed how to leave the study if they wished, which they could do at any time. Verbal consent was obtained at the beginning and end of the focus group to proceed with the focus group and analysis, respectively. Focus group participants were reminded of confidentiality at the beginning and the end of the focus group.

## Results

A total of 118 health workers participated in the questionnaire phase, conducted between June and December 2023. The focus group discussion followed in November 2024, with 7 participants representing a total of 29,800 health workers and students, selected from various Kenyan professional healthcare associations, including their student and young professional networks.

### Results phase I: Questionnaire

#### Demographics

**Figure 1.**
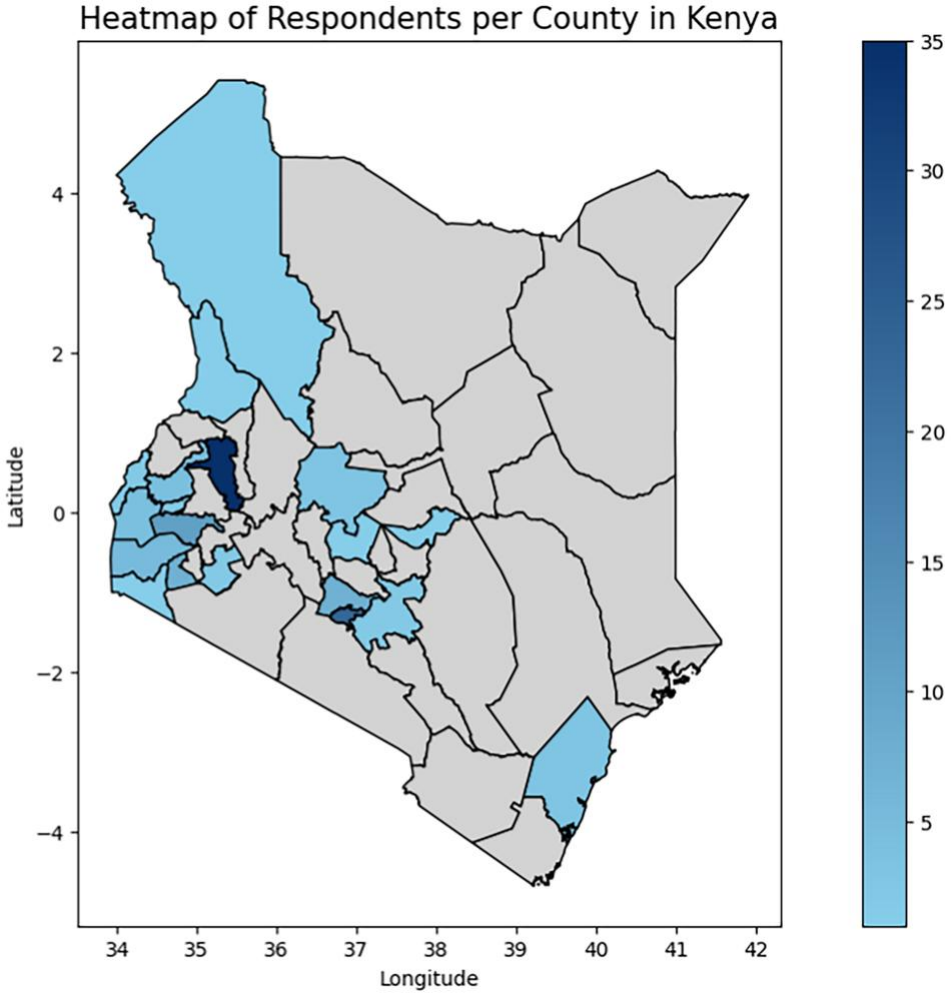
Heat map of Kenya presenting counties in which questionnaire respondents work primarily.

Of the 118 participants in the questionnaire, 67 (56.8%) were practising health professionals, including junior doctors, general practitioners, and specialists, while 51 (43.2%) were students training as health professionals, primarily in medical, nursing, and pharmacy fields. Medical doctors made up 24 participants (20.3%), with nurses and nursing students accounting for 8 participants (6.8%). Other professions included pharmacists, community health workers, microbiologists, and public health officers. Participants worked and studied in 20 counties, with the largest groups in Uasin Gishu (29.7%, *n* = 35), Nairobi (19.5%, *n* = 23), Kisumu (9.3%, *n* = 11), and Kiambu (5.9%, *n* = 7) (see Fig. 1). Most respondents (40.7%, *n* = 48) were active in public healthcare, while 16.1% (*n* = 19) were in private facilities, 11.0% (*n* = 13) in NGO-based providers, and 4.2% (*n* = 5) in faith-based institutions. 47.5% (*n* = 56) of participants were women and 52.5% (*n* = 62) were men. Ages ranged from 19 to 57 years, with a mean age of 27.2 years. The majority of participants (75%, *n* = 88) were aged 20–30.

Compared to available data on the Kenyan healthcare workforce, which is predominantly young and includes approximately 58% women and 42% men, the sample is reasonably representative in terms of gender but skews toward younger participants due to the inclusion of students. Geographically, the participation aligns with known trends of higher workforce concentrations in urban areas, though some underrepresentation of rural counties is noted [11].

#### Knowledge & experience

Respondents rated their knowledge of climate change and health at a mean of 6.84 (SD: 2.24) on a scale of 1 to 10, indicating a perception of moderate knowledge. Most participants viewed climate change as a major threat to health, with 60% strongly agreeing and 30% agreeing. Similarly, 80% of respondents strongly agreed (45%, *n* = 53) or agreed (35%, *n* = 41) to having witnessed the effects of climate change in their practice. Greenhouse gas emissions (55%, *n* = 65 strongly agreeing, 30%, *n* = 35 agreeing) and air pollution (65%, *n* = 77 strongly agreeing and 25%, *n* = 30 agreeing) were recognized as a significant health threats.

#### Perceptions of the healthcare system’s role in emission reduction and climate change mitigation

Opinions on the healthcare system’s current efforts in reducing GHG emissions were mixed, with 40% (*n* = 47) agreeing or strongly agreeing this was taken into consideration, while 35% (*n* = 41) disagreed or strongly disagreed. However, 90% (*n* = 106) agreed that reducing GHG emissions should be integrated into healthcare practices.

Regarding Kenya’s goal of a net-zero healthcare system by 2030, 45% (*n* = 53) agreed it as achievable, while 25% (*n* = 30) disagreed. There was strong support for the role of health workers, with 90% (*n* = 106) agreeing that they should lead advocacy and implementation efforts to reduce emissions.

Environmental concern was high, with 95% (*n* = 112) of respondents agreeing the current state is alarming, and just as many expressing an interest in learning how to reduce GHG emissions in healthcare. Responsibility was seen as shared, with 95% (*n* = 112) agreeing that the government and the private sector should take responsibility, and 90% (*n* = 106) supporting roles for community leaders and individuals.

#### Sources of healthcare emissions and current interventions in emission reduction

The majority of respondents (84%, *n* = 99) identified the production, transport, and disposal of goods and services—such as pharmaceuticals, medical devices, and hospital equipment (emission scope 3 emissions)—as the largest contributor to emissions in Kenya’s healthcare system. Additionally, 10% pointed to indirect emissions from purchased energy sources, such as electricity, steam, cooling, and heating (scope 2), while 4% highlighted emissions directly from healthcare facilities and vehicles (scope 1).

Regarding actions taken to reduce greenhouse gas emissions, 87 respondents (74%) reported that they have not yet implemented any interventions. However, some respondents have engaged in efforts like waste management, recycling, energy efficiency measures (e.g. solar power), and sustainable transportation. Education and advocacy were also frequently mentioned as key opportunity areas of focus for reducing emissions. A large proportion of respondents (95%, *n* = 112) expressed interest in implementing future interventions, such as tree planting, better waste management, and using alternative energy sources.

#### Proposed solutions

Respondents identified several key interventions to reduce greenhouse gas emissions in Kenya’s healthcare system. The most frequently mentioned intervention was the adoption of renewable energy sources (e.g. solar and wind) for healthcare facilities to decrease reliance on fossil fuels. In addition, supply chain management strategies, such as proper disposal of medical waste, increased recycling, and minimizing single-use products, were widely supported. Respondents also advocated for telemedicine as a means to reduce patient travel and associated transportation emissions. Other recurring suggestions included sustainable transportation initiatives, such as adopting electric vehicles and encouraging carpooling or public transport, and education and awareness programs aimed at health workers and the general public to promote sustainable practices. Finally, respondents emphasized the importance of green procurement, focusing on the purchase of eco-friendly, recyclable, and energy-efficient products.

Participants highlighted the critical need for integrating climate change adaptation into Kenya’s healthcare system, with a strong focus on emergency preparedness and resilient infrastructure. This includes retrofitting facilities to withstand extreme weather events and ensuring reliable energy systems powered by renewable energy sources such as solar panels. In addition, respondents emphasized the importance of telemedicine to reduce travel and maintain continuity of care during climate disruptions, which also aligns with the broader strategy to reduce emissions. Building sustainable supply chains was also viewed as a key opportunity to reduce emissions and adapt, through promoting the use of locally sourced materials.

#### Opportunities & barriers

Several opportunities for successfully implementing these measures were identified. Policy and regulatory frameworks were considered essential to encourage healthcare facilities to prioritize sustainability. Many respondents saw public-private partnerships as a key opportunity for mobilizing funding and resources to support emission reduction initiatives. Technological innovation, such as energy-efficient medical devices and advanced waste disposal systems, was viewed as another critical factor in driving progress. Additionally, community engagement—including tree-planting campaigns and public awareness programs—was frequently mentioned as a way to promote sustainability at the local level.

The most significant barrier identified by respondents was financial constraints, particularly the lack of funding for the adoption of green technologies and waste management infrastructure. Lack of awareness and education among health workers and the public was also seen as a major obstacle. Other barriers included resistance to change within healthcare institutions and infrastructure limitations, with some facilities lacking the capacity to implement renewable energy or waste management systems.

To overcome these barriers, respondents recommended increased funding and financial incentives, such as government grants or international donor support, to facilitate the transition to greener technologies. Education and training programs were seen as crucial to raising awareness and addressing resistance to change. Respondents also called for stronger policy enforcement to compel healthcare facilities to adopt emission reduction measures. Finally, they highlighted the importance of collaboration and partnerships between government, healthcare institutions, and environmental organizations to support the implementation of sustainable practices.

Finally, when asked whether Kenya needs to change its approach to zero emissions of the healthcare system if it is going to be successful, respondents overwhelmingly called for stronger policies and better enforcement. Key suggestions included prioritizing renewable energy adoption, improving waste management practices, and increasing government investment in climate-resilient infrastructure. Education and capacity-building initiatives for health workers and public awareness campaigns were seen as critical to driving change. Additionally, multisectoral collaboration, public-private partnerships, and international cooperation were identified as essential for securing the necessary funding and technological innovation to achieve zero emissions in the healthcare system.

### Results phase II: Focus group on education

The findings from Phase I highlighted that while health workers are seen by key stakeholders and decisionmakers as key drivers in promoting sustainability and resilience of the healthcare system, many still lack the necessary education and training to effectively fulfil this role. A focus group was conducted to explore how education might equip health workers with the knowledge and skills needed to lead in implementing emission reduction strategies and climate adaptation within the healthcare system.

A total of seven representatives from professional and student organizations participated, including four women and three men. Collectively, they represented over 29,800 health workers and university students, including community health workers, dentists, pharmacists, nurses, medical doctors, family physicians, and pharmacy and medical students. Participants brought a wide range of perspectives, spanning clinical, educational, and advocacy roles within the healthcare system. The second facilitator noted that participants engaged openly and confidently, with no evident cultural or contextual barriers influencing the discussion.

The discussion began by validating the outcomes of the questionnaire, confirming that while awareness about climate change among health workers is generally high, there is a significant gap in actionable knowledge and practical skills. One participant reflected this sentiment by referencing a similar internal survey:

“The majority know that climate change is there and impacting the work, but there is very little knowledge about what has been done or what can be done.” (Participant 5, Representing Medical Practitioners, Pharmacists, and Dentists)

This lack of practical knowledge is further compounded by the increasing burden on health workers due to emerging disease patterns linked to climate change. One participant shared a vivid account of the challenges in a rural clinic, where a lack of preparedness for flooding led to delayed patient care, significant supply chain disruptions, and outbreaks of waterborne diseases. Another participant highlighted the strain on the health workers:

“There is an increased workload due to these new patterns and new diseases.” (Participant 5, Representing Medical Practitioners, Pharmacists, and Dentists)

Participants emphasized the dual role of health workers as both caregivers and advocates for climate and health. Beyond clinical responsibilities, they are deeply embedded in their communities, where they serve as trusted sources of knowledge and agents of change. One participant illustrated this by stating,

“Health workers are also health advocates for the communities in which they live. So, educating one single health worker from a community is an immense opportunity to addressing some of the issues that we have talked about.” (Participant 5, Representing Medical Practitioners, Pharmacists, and Dentists)

However, a disconnect between national policies and local realities was consistently noted. Participants felt that while national policies like the National Climate Change Action Plan outline ambitious goals, their relevance and applicability to local contexts remain unclear. The group strongly recommended bridging this gap by tailoring policy implementation to reflect the lived realities of health workers and the communities they serve, and ensuring funding is allocated to national plans. One participant remarked,

“Family physicians transcend between the facility and the community, but how national policies and information is distilled for action or awareness downstream to us remains vague.” (Participant 3, representing Family Physicians)

The focus group also identified several key gaps in education. These included education on climate change and health overall, training on disaster response, managing shifts in disease burden, and integrating sustainability into healthcare practices. Participants stressed the importance of a generic teaching framework during university and for working professionals that allows for contextualization to local realities, ensuring the training is adaptable and relevant. They emphasized the need for practical, actionable education that equips health workers with the skills to address these challenges effectively, while also fostering their ability to disseminate critical health information to communities, including in local languages. In addition to practical education, participants highlighted the need for professional development that fosters emotional resilience and equips health workers to navigate the ethical challenges of addressing climate change. Reflective practice and advocacy emerged as essential competencies, enabling health workers to lead in their communities while maintaining their well-being amidst crises.

Young professionals and students emerged as a critical group, with participants highlighting their heightened awareness of climate-health issues and their potential as change agents. One participant noted,

“The younger generation is more knowledgeable about climate and health, which is a privilege we older colleagues do not have.” (Participant 2, Representing Young Doctors)

This was accompanied by recognition of their challenges, particularly limited access to decision-making processes and resources. Participants stressed the importance of empowering these groups through targeted education, mentorship, and leadership opportunities to enable them to contribute effectively to sustainability efforts. As one participant emphasized,

“We need to ensure that young professionals and students have the tools and platforms to translate their enthusiasm into actionable change.” (Participant 1, Representing Medical Students)

Finally, the group proposed a range of recommendations for improving climate-health education. They underscored the importance of co-creation and decolonization in designing educational programs, guided by the principle of “*nothing for us without us*”, which prioritizes community involvement, partnership, and building local assets. Participants highlighted the value of involving diverse stakeholders, including environmentalists, universities, trade unions, tertiary colleges, religious institutions, civil society organizations, county assemblies, county departments of environment and climate change, the Ministry of Health, and international bodies such as the United Nations, in addition to engaging communities at local levels to ensure alignment with both (inter)national policies and grassroots needs. Creative approaches to education were also suggested, such as leveraging social media, facilitating knowledge exchange through peer-led workshops, and embedding climate-health modules within existing curricula. The participants stressed that such strategies must remain community-focused, inclusive, and empowering, ensuring health workers can actively engage with and address the needs of the populations they serve. One participant reinforced the value of research in advancing actionable insights for climate-health education, noting:

“Research such as this is needed.” (Participant 5, Representing Medical Practitioners, Pharmacists, and Dentists)

## Discussion

Climate change poses significant challenges to healthcare systems worldwide, particularly in low- and middle-income countries like Kenya, where health workers are grappling with the dual responsibility of mitigating emissions while adapting to climate impacts [12]. This study offers a unique perspective on the perceptions, knowledge, and roles of Kenyan health workers as the country transitions toward a net-zero, climate-resilient healthcare system. In alignment with global goals such as the Paris Agreement and Kenya’s commitments under the World Health Organization’s Health Programme at COP26, this research highlights both the opportunities and barriers that health workers encounter as key stakeholders in these efforts [13, 14].

The questionnaire responses of Kenyan health workers reveal a generally high level of concern about climate change, with 90% of respondents acknowledging the importance of integrating GHG emissions reduction into healthcare practices. This strong consensus reflects the global recognition that healthcare systems must play a central role in combating climate change, not only because of their direct emissions but also due to the public health threats posed by climate-related disruptions [15]. Similarly, the identification of supply chain emissions as the largest contributor to healthcare’s carbon footprint aligns with global estimates that have demonstrated the outsized impact of procurement and product usage in hospitals [2].

A noteworthy finding is the widespread support for renewable energy adoption as a key solution to reduce emissions, a sentiment echoed in other LMICs, where renewable energy presents a cost-effective and sustainable alternative to traditional energy sources in healthcare [16]. The emphasis on telemedicine as a means of reducing travel-related emissions is also consistent with global trends whereby it has gained significant traction during the COVID-19 pandemic and has been advocated as a sustainable model for future healthcare delivery [17].

The barriers identified in this study, particularly financial constraints and the limited integration of climate-health topics in existing education systems, align with findings from similar contexts. The perception of a lack of enforcement of policy frameworks is another recurrent theme that has been widely documented in both global and national studies. Health workers in Kenya highlighted the need for stronger governmental leadership and more effective policy implementation, echoing calls for healthcare policies that are better integrated with national climate strategies [18]. While Kenya’s contribution to global emissions is minimal, the strong perception among participants that national governments and private sector actors hold primary responsibility for mitigation and adaptation likely reflects their central role in enabling change within the Kenyan healthcare system. This perspective also underscores participants’ alignment with national policies such as the National Climate Change Action Plan and Kenya’s commitments under the WHO COP26 Health Programme. At the same time, it highlights the need for international support, such as funding, technology transfer, and educational partnerships, to ensure such commitments are realised. Health workers’ “dual responsibility” to mitigate and adapt thus reflects not only their willingness to lead change, but also their recognition of systemic dependencies that span national and global levels. Framing climate-health education as an adaptation and mitigation measure provides a compelling entry point for international collaboration, particularly with institutions in high-emitting countries that bear a historic responsibility and are well positioned to support transformative education and capacity-building efforts. Furthermore, the call for multisectoral collaboration and international cooperation aligns with recommendations from the World Health Organization and its Alliance for Transformative Action on Climate and Health (ATACH), which underscores the importance of cross-sector partnerships in achieving climate-resilient health systems [19].

An assessment in 2022 showed that South African health workers, despite positive attitudes towards environmental sustainability, lacked the necessary knowledge and training to implement effective practices [20]. Like our findings, this emphasizes the critical need for targeted education and capacity-building to empower health workers to lead sustainability efforts. Without such educational initiatives, progress towards sustainable healthcare will remain limited, underscoring the urgency of integrating climate education into healthcare training.

Through the questionnaire, education emerged as a cornerstone in achieving sustainable, resilient healthcare. This emphasis on education may be influenced by the high proportion of student participants, whose active engagement in learning may have heightened their awareness of educational gaps. Their dual identity as emerging professionals and current learners brings valuable insight into the urgent need for climate-health training. The focus group then further validated global assertions that healthcare education must transition from traditional disease-focused approaches to include sustainability as a core component [21, 22]. In Kenya, the focus group participants emphasized a disconnect between national policy ambitions and local realities, underscoring the need for education that bridges this gap. This aligns with the literature advocating for systems thinking and context-specific approaches to training, ensuring that policies are actionable and resonate with the lived realities of health workers [22].

Building on this, integrating sustainable healthcare education in Kenya requires a transformative approach that prioritizes contextual relevance and societal impact. This need for transformation is highlighted in the focus group findings, which identified critical gaps in practical knowledge and skills, particularly in translating policy into actionable local strategies. Transformative learning, as adapted by Redvers from Freire’s pedagogy, goes beyond traditional methods by embedding principles of societal change, advocacy, and justice. This approach aligns with the gaps identified by our participants, particularly in addressing the disconnection between policy and practice. Transformative education necessitates interdisciplinary, place-based, and action-oriented learning that integrates personal and collective experiences, empirical observation, and an ethico-political understanding of both local and global relevance [23, 24].

Participants in the focus group reinforced the principle of “nothing for us without us,” advocating for educational co-creation with communities and stakeholders towards decolonization of health education. This aligns well with global transformative education frameworks emphasizing the inclusion of Indigenous and local knowledge systems as critical to planetary health solutions [22, 23]. Incorporating local languages and community-driven approaches improves inclusivity and empowers health workers to act as advocates and educators within their own contexts.

Rooted in praxis, transformative education bridges knowledge and action, fostering critical thinking and relational care. This includes co-creating curricula with communities, emphasizing place-based and experiential learning, and incorporating diverse knowledge systems, such as Indigenous perspectives. The principles of compassion, knowledge, and reflection central to this educational model enable health workers to navigate and address the profound challenges posed by climate change, positioning them as advocates and agents of social and environmental justice. Additionally, embedding sustainability into healthcare education must consider the interconnectedness of ecological, social, and health systems. Practical implementation requires curricular integration of sustainability concepts and the cultivation of values that inspire future healthcare professionals to lead meaningful systemic change [22, 23].

From a practical standpoint, the focus group proposed both formalized and informal strategies for integrating climate-health education into existing systems. Formalized approaches included embedding sustainability modules within existing health curricula, ensuring alignment with national climate policies, and developing structured, recognized educational programs as part of healthcare worker development initiatives. Informal strategies focused on utilizing social media to disseminate knowledge and increase accessibility, as well as fostering experiential learning and knowledge exchange through self-organized, peer-led workshops. These approaches collectively echo the emphasis in sustainable healthcare education literature on embedding sustainability across curricula and leveraging digital tools for widespread impact [21, 22].

Finally, as health workers navigate the challenging realities of climate change, emotional resilience and ethical leadership were recognized as integral to education. The literature underscores the role of reflective practice and advocacy as essential competencies for health workers, particularly in LMICs, where resource constraints often magnify challenges [22]. Young professionals and students, with their heightened awareness of climate-health issues and openness to innovation, were identified as pivotal change agents. However, systemic barriers, such as limited access to leadership roles, hinder their ability to drive meaningful change. By prioritizing capacity-building through education, healthcare systems can not only empower individuals but also enhance their overall resilience and ability to address climate-related challenges effectively.

### Strengths & limitations

This study offers valuable insights into the perceptions and roles of Kenyan health workers in climate mitigation and adaptation, contributing to a growing body of research on sustainable, resilient healthcare. A key strength lies in its mixed-methods approach, which allowed for an exploration of both broad trends through the questionnaire and deeper contextual insights via the focus group. The recruitment of participants through professional and student healthcare associations ensured diverse representation across a range of professions, healthcare settings, and regions. Additionally, participant checking of questionnaire findings in the focus group strengthened the credibility and validity of the results.

However, several limitations must be acknowledged. Convenience sampling was used for the questionnaire, relying on participants’ availability and willingness to engage. While this method is well-suited for exploratory studies like this, it may have introduced selection bias, potentially overrepresenting individuals with a pre-existing interest in climate change. Consequently, the findings may not fully reflect the views of the broader Kenyan health workforce. The reliance on online recruitment and data collection may have further excluded participants from underserved or remote areas with limited internet access, affecting the representativeness of the sample. Moreover, the questionnaire relied on self-reported knowledge of climate and health issues rather than explicitly testing this knowledge. This may have resulted in participants overestimating or underestimating their actual level of knowledge, adding potential bias to the findings. Further, the number of participants per professional group was small, limiting the ability to draw profession-specific conclusions. While many participants expressed strong willingness to engage in sustainable practices, the study did not assess whether such willingness would translate into behavioral change in clinical practice. Future research could explore implementation further.

The focus group employed purposive sampling to gather diverse perspectives from key healthcare stakeholders. While this approach enabled rich qualitative insights, the small sample size and reliance on association representatives may not fully capture the experiences of health workers in all contexts. Additionally, the virtual format of the focus group, while pragmatic given geographic constraints, may have limited opportunities for informal interaction or non-verbal communication, which are often more readily observed in in-person discussions.

It is also important to acknowledge the positionality of the research team. While MO, a Kenyan researcher, played a central role in contextualizing the study and ensuring cultural relevance, the lead researcher from the global north (IMB) may still represent perceived power imbalances in conducting research in a middle-income country. Efforts were made to mitigate this by incorporating input from Kenyan collaborators throughout the study design, data collection, and interpretation.

Despite these limitations, this study provides foundational insights into the educational and policy needs of Kenyan health workers in the context of climate change. Future research should aim to address these limitations by employing broader recruitment strategies, combining virtual and in-person methodologies, and expanding participant representation to capture a wider range of perspectives.

### Conclusion

This study highlights the pivotal role of health workers in Kenya’s transition to a net-zero, climate-resilient healthcare system. Education emerges as a cornerstone in bridging the gap between policy ambitions and actionable practices, addressing critical barriers such as limited knowledge and the disconnect between national strategies and local realities. By equipping health workers with practical skills, reflective capacities, and systemic understanding, transformative education provides a pathway to empower them as leaders in sustainable healthcare.

Currently, the WHO’s ATACH presents an opportunity to incorporate an educational focus within its framework. Integrating transformative education into ATACH’s goals can address the complex interconnections between health, climate, and equity, equipping health workers with the necessary tools to advocate for and implement meaningful change. Transformative education has the potential to catalyse systemic change, fostering a health workforce that is not only prepared to meet current challenges but also to lead the way in creating equitable, sustainable solutions for future generations.

By investing in education that prioritizes contextual relevance, societal impact, and collaboration, Kenya can ensure that its healthcare system evolves into a model of climate resilience and sustainability, with health workers at the forefront of this critical transformation.

## Supplementary Material

kgaf026_Supplementary_Data

## Data Availability

The data underlying this article will be shared on reasonable request to the corresponding author. The information sheet, informed consent form and focus group topic guide are available in the Supplementary Information.
