# Supplementary material for: The role of health workers in Kenya’s Net-Zero transition: a Mixed-Methods study on healthcare system climate change mitigation and adaptation
Source: Oxf Open Clim Chang. 2025 Dec 1;5(1):kgaf026. doi: 10.1093/oxfclm/kgaf026 (PMC12681247; doi:10.1093/oxfclm/kgaf026)
Supplement: kgaf026_Supplementary_Data [file kgaf026_supplementary_data.zip › Supplementary Information.docx]

Supplementary Information to The Role of Health Workers in Kenya’s Net-Zero Transition: A Mixed-Methods Study on Healthcare System Climate Change Mitigation and Adaptation

Iris Martine Blom MD (0000-0002-1294-7408), Melvine Anyango Otieno MSc (0000-0001-9241-9180), Marie-Claire Wangari MBChB (0000-0002-4529-8133), Agan Leonard MSc (0009-0004-2070-8118), Winslet Mwende (0009-0000-5588-5166), Naomi Wanjiku Gitau (0009-0006-4988-2456), Iain Cross PhD (0000-0002-8245-5628), Anita Berlin PhD (0000-0002-9745-5735), Andy Haines F Med Sci (0000-0002-8053-4605), Sarah Whitmee PhD (0000-0001-9161-868X)

Appendix I Full Questionnaire

**Participant Information Sheet
Title of Project**: Greenhouse gas mitigation of the Kenyan health care system

**Introduction**We would like to invite you to take part in a research study. Joining the study is entirely up to you. Before you decide, you need to understand why the research is being done and what it would involve. Ask questions if anything you read is not clear or you would like more information. Please feel free to talk to others about the study if you wish. Take time to decide whether or not to take part.

**What is the purpose of the study?**Climate change is having and is increasingly expected to have direct and indirect effects on health. Health care systems should adapt to deal with these effects, whilst mitigating their own greenhouse gas emissions to not further contribute to the health emergency. This poses an opportunity for health care systems to advance in an environmentally sustainable manner. A key challenge is to identify pathways towards advancement whilst mitigating emissions in health care systems, with a particular evidence gap in Low- and Middle-Income Countries.

The London School of Hygiene and Tropical Medicine (LSHTM) are conducting research on greenhouse gas mitigation of health care systems and particularly in Kenya to identify lessons learned, pathways, opportunities, barriers and solutions towards greenhouse gas mitigation of health care systems and through that create recommendations for more environmentally sustainable health care.

**Why have I been asked to take part?**You have been invited because we believe your knowledge, experiences and expertise will contribute to a better understanding of this topic and we would like to build our research and recommendations on your guidance.

**Do I have to take part?**No. It is up to you to decide to take part or not. If you don’t want to take part, that’s ok.

**What will I have to do?**Participate in a questionnaire which will take approximately 10 minutes to fill out.

**What are the possible risks and disadvantages?**Because this study only involves participating in a questionnaire we do not anticipate any harm or discomfort for you other than the time it will take you to participate in the questionnaire. There are two potential risks with a low likelihood of occurring, which are information risks (e.g., loss of privacy and/or breach of confidentiality), for which a data management plan is in place, ethical approval and data regulation is sought, and all researchers carry legal responsibility to minimize this risk as much as possible, and psychological or emotional risks (e.g., fear, stress, confusion, guilt) which will be minimized by information provision and contact information for any questions.

**What are the possible benefits?**We cannot promise the study will help you but the information we get from the study will help our knowledge and understanding of this research area of greenhouse gas mitigation of health care systems.

**What if something goes wrong?**If you have a concern about any aspect of this study, you should ask to speak to the researchers who will do their best to answer your questions: 00000000000 / 0000000000. If you remain unhappy and wish to complain formally, you can do this by contacting: 00000000000 at 00000000 or 0000000000.

The London School of Hygiene and Tropical Medicine holds insurance policies which apply to this study. If you experience harm or injury as a result of taking part in this study, you may be eligible to claim compensation.

**Can I change my mind about taking part?**You can withdraw from the study at any time. If you wish to withdraw we will securely keep your recorded contribution up to the point of withdrawal. If you withdraw, you can choose to still include your contribution in the anonymised transcript or request that we remove all your comments from the final transcript and written records. If you withdraw, you can choose whether we retain your personal information after withdrawal or remove it from our records.

**What will happen to information collected about me?**We will need to use information from you. All information collected about you will be kept private. Only the study staff and authorities who check that the study is being carried out properly will be allowed to look at information about you. Information will include your name, , gender, age, contact details and position. We will keep all information about you safe and secure.

Data may be sent to other study staff in London but this will be anonymised. This means that any information about you which is shared beyond the chief investigator and the two supervisors, will have your name and address removed so that you cannot be recognised and your data will have a code number instead.

Your personal details, meaning your name and other identifiable information, will be kept in a different safe place to the other study information and will be destroyed within 10 years of the end of the study.

At the end of the project, the study data will be archived at the data compass at the London School of Hygiene & Tropical Medicine. The data will be made available to other researchers worldwide for research and to improve medical knowledge and patient care. Your personal information will not be included and there is no way that you can be identified.

**What will happen to the results of this study?**The study results will be published in a medical journal so that other researchers and policymakers can learn from them. Your personal information will not be included in the study report and there is no way that you can be identified from it.

**Who is organising and funding this study?**London School of Hygiene & Tropical Medicine is the sponsor for the research and they have full responsibility for the project including the collection, storage and analysis of your data, and will act as the Data Controller for the study. This means that we are responsible for looking after your information and using it properly.

Funding for the study has been provided by the Prince Bernhard Culture Fund, Stichting VSBFonds and the dr. Hendrik Mullerfonds. The funders play no role in study design, conduct, data analysis and interpretation, manuscript writing, and dissemination of results.

**Who has reviewed this study?**
All research involving human participants is looked at by an independent group of people, called a Research Ethics Committee, to protect your interests. This study has been reviewed and given favourable opinion by The London School of Hygiene and Tropical Medicine Research Ethics Committee (28210). The Kenya Medical Research Institute approved the study (4662) and the National Commission for Science, Technology and Innovation provided a license (519115).

**Further information and contact details**
Thank you for taking time to read this information sheet. If you think you will take part in the study please read and sign the consent form on the next page of the form.

If you would like any further information, please contact Dr Iris Martine Blom who can answer any questions you may have about the study.

Contact details:
Dr Iris Martine Blom
Email: 0000000000000000
Telephone: 000000000000

If you have any concerns about the study please contact the head of research governance at LSHTM 000000000000 at 0000000000 or 000000000.

**Consent Form**

Please write your full name below* [* indicates mandatory question]

Your answer

*

- I have read the written information OR
- I have had the information explained to me by study personnel in a language that I understand,

and I*

Confirm

- confirm that my choice to participate is entirely voluntarily,
- confirm that I have had the opportunity to ask questions about this study and I am happy with the answers that have been provided,
- understand that I allow access to the information about me by the persons described in the information sheet,
- agree to be quoted anonymously in the study findings,
- agree for anonymised data from my questionnaire to be stored at the London School of Hygiene & Tropical Medicine until all study outputs are completed, and shared with other researchers on request in future
- had enough time to think about whether I want to take part in this study,
- agree to take part in this study.
- confirm that my choice to participate is entirely voluntarily,
- confirm that I have had the opportunity to ask questions about this study and I am happy with the answers that have been provided,
- understand that I allow access to the information about me by the persons described in the information sheet,
- agree to be quoted anonymously in the study findings,
- agree for anonymised data from my questionnaire to be stored at the London School of Hygiene & Tropical Medicine until all study outputs are completed, and shared with other researchers on request in future
- had enough time to think about whether I want to take part in this study,
- agree to take part in this study.

**Background Information**

Are you a health professional or student?*

- Health professional
- Student

Do you work or study in Kenya?*

- Yes

What is your primary health profession or study? *

- Medical doctor: Specialist
- Family doctor
- Medical doctor: junior doctor
- Medical doctor: resident
- General practicioner
- Medical student
- Nurse
- Nurse practitioner
- Nursing student
- Physiotherapist
- Physiotherapy student
- Nutritionist
- Nutrition student
- Midwife
- Midwifery student
- Dentist
- Dentistry student
- Dietitian
- Dietitian student
- Community health care worker
- Pharmacist
- Pharmacy student
- Other:

What is your specialisation?*

- Choose

What County do you primarily work in now? *

- Choose

What type of healthcare provider do you work for?*

- Public healthcare provider (e.g. national, county, sub-county hospitals)
- Private healthcare provider (e.g. AAR, Aga Khan, MP Shah)
- Faith-based healthcare provider (e.g. Kijabe, Tenwek, Matter)
- NGO-based healthcare provider (e.g. Amref Clinics)
- Other:

What is your sex?*

- Male
- Female

What is your age?*

- Choose

Would you like to receive a summary of the outcomes of this study?*

- Yes
- No

What is your email address? If you answered yes to the previous question, please note down your email address here which will be used to share the summary of the outcomes of the study with you.

Your answer

**Climate change & health**

On a scale from 1 to 10, how would you rate your knowledge of climate change and health?*

Very limited knowledge (1) - Very extensive knowledge (10)

Please rate how much you agree with the following statements:*

Strongly Disagree – Disagree - Somewhat Disagree - Neither Agree nor Disagree - Somewhat Agree – Agree - Strongly Agree

- Climate change is a major threat to health.
- I witness the effects of climate change on health in my practice.
- Greenhouse gas emissions are a major threat to health.
- Air pollution is a major threat to health.
- Climate change is a major threat to health.
- I witness the effects of climate change on health in my practice.
- Greenhouse gas emissions are a major threat to health.
- Air pollution is a major threat to health.

**Practice**

Please rate how much you agree with the following statements:*

Strongly Disagree – Disagree - Somewhat Disagree - Neither Agree nor Disagree - Somewhat Agree – Agree - Strongly Agree

- The healthcare system is currently taking reducing greenhouse gas emissions into consideration in healthcare practices.
- Kenya can achieve its commitment to a net-zero healthcare system by 2030 (this means that the net amount of greenhouse gas emissions added to the atmosphere by the healthcare system is zero).
- Reducing greenhouse gas emissions should be incorporated into healthcare practices.
- Health workers should take a leading role in advocating for reducing greenhouse gas emissions in the healthcare system.
- Health workers should take a leading role in implementing the reduction of greenhouse gas emissions in the healthcare system.
- The current state of our environment (including the rate of climate change) is concerning.
- I am interested in learning how to reduce greenhouse gas emissions in my healthcare practice.
- The government has to take responsibility in terms of reducing greenhouse gas emissions in the healthcare system.
- The private sector (producers, pharmaceutical companies, etc.) has to take responsibility in terms of reducing greenhouse gas emissions in the healthcare system.
- Health workers have to take responsibility in terms of reducing greenhouse gas emissions in the healthcare system.
- Leadership in communities (counselors, chiefs etc.) has to take responsibility in terms of reducing greenhouse gas emissions in the healthcare system.
- Individuals have to take responsibility in terms of reducing greenhouse gas emissions in the healthcare system.
- The healthcare system is currently taking reducing greenhouse gas emissions into consideration in healthcare practices.
- Kenya can achieve its commitment to a net-zero healthcare system by 2030 (this means that the net amount of greenhouse gas emissions added to the atmosphere by the healthcare system is zero).
- Reducing greenhouse gas emissions should be incorporated into healthcare practices.
- Health workers should take a leading role in advocating for reducing greenhouse gas emissions in the healthcare system.
- Health workers should take a leading role in implementing the reduction of greenhouse gas emissions in the healthcare system.
- The current state of our environment (including the rate of climate change) is concerning.
- I am interested in learning how to reduce greenhouse gas emissions in my healthcare practice.
- The government has to take responsibility in terms of reducing greenhouse gas emissions in the healthcare system.
- The private sector (producers, pharmaceutical companies, etc.) has to take responsibility in terms of reducing greenhouse gas emissions in the healthcare system.
- Health workers have to take responsibility in terms of reducing greenhouse gas emissions in the healthcare system.
- Leadership in communities (counselors, chiefs etc.) has to take responsibility in terms of reducing greenhouse gas emissions in the healthcare system.
- Individuals have to take responsibility in terms of reducing greenhouse gas emissions in the healthcare system.

**Measures**

In your opinion, which part of the health care system causes the highest emissions in Kenya?*

- Emissions emanating directly from healthcare facilities and health care owned vehicles.
- Indirect emissions from purchased energy sources such as electricity, steam, cooling, and heating.
- The production, transport, and disposal of goods and services, such as pharmaceuticals and other chemicals, food and agricultural products, medical devices, hospital equipment, and instruments.

Have you already implemented interventions to reduce greenhouse gas emissions in your health practice? If so, which ones?*

If so, please use the 'other' box to type yes and explain how.

- No
- Other:

Are you planning to implement interventions to reduce greenhouse gas emissions in your health practice? If so, which ones?*

If so, please use the 'other' box to type yes and explain how.

- No
- Other:

Are you interested to implement interventions to reduce greenhouse gas emissions in your health practice?*

- Yes
- No

What are your ideas for interventions that should be implemented to reduce greenhouse gas emissions in the healthcare system in Kenya?*

**Opportunities & Barriers**

Are there key opportunities to implement successful measures to reduce greenhouse gas emissions in the health care system? If so, what are they?*

Your answer

How can these opportunities be used to the best advantage?*

Your answer

Are there key barriers to implementing successful measures to reduce greenhouse gas emissions in the healthcare system? What are they?*

Your answer

How can these barriers be overcome?*

Your answer

How are or should these measures be funded?*

Your answer

**Adapting to the impact of climate change**

To deal with the impacts of climate change, the healthcare system needs to transform and adapt to these impacts. How can this adaptation be considered when reducing greenhouse gas emissions in the healthcare system?*

Your answer

Is adapting the healthcare system currently being considered and if so, how?*

If so, please use the 'other' box to type yes and explain how.

- No
- Other:

**General**

Does Kenya need to change its approach to zero emissions of the healthcare system if it is going to be successful? If so, what does it need to do? *

If so, please use the 'other' box to type yes and explain how.

- No
- Other

Appendix II Focus Group Topic Guide

**Session Duration:** 2.5 hours

**1. Welcome and Introduction (15 minutes)**

- Welcome participants and outline the session objectives.
- Present questionnaire outcomes (5 minutes): Provide a brief overview of key findings, focusing on identified knowledge gaps, barriers, and opportunities for action.
- Share the NCCAP document (briefly explain its significance).
- Review focus group structure, emphasizing confidentiality and the value of their contributions.
- Facilitate introductions: Ask each participant to state their role and their organization.

**2. Framing the Discussion: Setting the Context (20 minutes)**

- Prompt 1: Are you familiar with these relevant policies and commitments at the national level and their relevance to healthcare?
  - Follow-up: Do you feel ownership of these goals in your role? Why or why not?
- Prompt 2: Climate-related health challenges are becoming more pressing globally and locally. What specific challenges have you faced in this area within your work?

**3. Exploring Knowledge, Skills, and Behaviours (30 minutes)**

- Prompt 3: What specific knowledge or skills do you feel are most urgently needed for health workers to address climate-related health challenges?
  - Follow-up on Practicality: And are there specific resources or tools that would help? you apply these skills?
  - Ask about examples: “Can you share situations where specific knowledge or skills were missing or made a difference?”

**4. Framework Exploration and Educational Needs (25 minutes)**

- Prompt 7: Education can help learners understand the interconnectedness between different levels:
  - Micro (individual): How personal action influences wider systems.
  - Meso (community): How communities act collectively and engage with individuals and institutions.
  - Macro (institutional): How institutions drive broader system-wide change.
  - Meta (universal): How global values and systems shape education for sustainability.
  - Discussion: In your experience, how can education for health workers foster understanding and action across these interconnected levels?

**5. Practical Solutions and Next Steps (30 minutes)**

- Prompt 8: If education or training were developed for health workers:
  - What should it focus on?
  - What should it look like?
  - Historically, healthcare education frameworks might be influenced by Western approaches. How can education for health workers better integrate local knowledge systems and community-specific practices?
- Prompt 9: Based on today’s discussion, what are the most practical steps we can take to improve climate-health education for health workers in Kenya? What actions can be taken at individual, organizational, and policy levels?

**6. Power Dynamics and Engagement (20 minutes)**

- Prompt 5: When it comes to implementing education or training on climate and health, what role do different stakeholders play (e.g., senior management, younger professionals, policymakers)?
- Prompt 6: Younger professionals and students often report being more engaged in issues around climate and health but having limited decision-making power. Does this play a role in efforts to integrate climate and health into health education and practice?

**7. Wrap-up and Reflection (10 minutes)**

- Summarize key themes and insights shared during the discussion.
- Open the floor for any final reflections or additional thoughts participants would like to share.
- Explain next steps: How the insights will inform further analysis, recommendations, or actions.
- Sharing contact details in case of any questions, comments or additions.
